# Public involvement in the priority setting activities of a wait time management initiative: a qualitative case study

**DOI:** 10.1186/1472-6963-7-186

**Published:** 2007-11-16

**Authors:** Rebecca A Bruni, Andreas Laupacis, Wendy Levinson, Douglas K Martin

**Affiliations:** 1 Joint Centre for Bioethics, University of Toronto, Toronto, Canada; 2 Department of Health Policy, Management and Evaluation, University of Toronto, Toronto, Canada; 3 Li Ka Shing Knowledge Institute, St. Michaels Hospital, Toronto, Canada; 4 Department of Medicine, University of Toronto, Toronto, Canada

## Abstract

**Background:**

As no health system can afford to provide all possible services and treatments for the people it serves, each system must set priorities. Priority setting decision makers are increasingly involving the public in policy making. This study focuses on public engagement in a key priority setting context that plagues every health system around the world: wait list management. The purpose of this study is to describe and evaluate priority setting for the Ontario Wait Time Strategy, with special attention to public engagement.

**Methods:**

This study was conducted at the Ontario Wait Time Strategy in Ontario, Canada which is part of a Federal-Territorial-Provincial initiative to improve access and reduce wait times in five areas: cancer, cardiac, sight restoration, joint replacements, and diagnostic imaging. There were two sources of data: (1) over 25 documents (e.g. strategic planning reports, public updates), and (2) 28 one-on-one interviews with informants (e.g. OWTS participants, MOHLTC representatives, clinicians, patient advocates). Analysis used a modified thematic technique in three phases: open coding, axial coding, and evaluation.

**Results:**

The Ontario Wait Time Strategy partially meets the four conditions of 'accountability for reasonableness'. The public was not directly involved in the priority setting activities of the Ontario Wait Time Strategy. Study participants identified both benefits (supporting the initiative, experts of the lived experience, a publicly funded system and sustainability of the healthcare system) and concerns (personal biases, lack of interest to be involved, time constraints, and level of technicality) for public involvement in the Ontario Wait Time Strategy. Additionally, the participants identified concern for the consequences (sustainability, cannibalism, and a class system) resulting from the Ontario Wait Times Strategy.

**Conclusion:**

We described and evaluated a wait time management initiative (the Ontario Wait Time Strategy) with special attention to public engagement, and provided a concrete plan to operationalize a strategy for improving public involvement in this, and other, wait time initiatives.

## Background

As no health system can afford to provide all possible services and treatments for the people it serves, each system must set priorities. The current rate of growth of health spending, which outpaces GDP growth in almost every country [1], is unsustainable. Therefore, priority setting is arguably the most prominent concern of health policy makers today.

Priority setting is fundamentally characterized by conflicts between competing values and there is no overarching theory to resolve these conflicts. Thus, Ham argues, "Given that that there is no right answer in the priority setting debate, an important justification for the decisions that are made is that they have been arrived as a result of due process [2]." Due process is characterized by legitimacy and fairness. Legitimacy refers to the moral authority of decision makers; fairness refers to conditions of the decision making process that render the outcomes morally acceptable. Legitimacy and fairness are related in that legitimacy may be enhanced by a commitment to fair decision making [3-6].

With these goals in mind, Daniels and Sabin developed, 'Accountability for Reasonableness' [7], a conceptual framework for legitimate and fair priority setting that has gained international recognition and emerged as the leading conceptual framework for priority setting researchers [5,8,9]. According to 'Accountability for Reasonableness' a fair priority setting process meets four conditions: relevance, publicity, revisions/appeals, and enforcement (described in Figure 2). 'Accountability for reasonableness' establishes a moral foundation for public involvement to enhance the legitimacy and fairness of priority setting, something that scholars and government reports have long advocated [10-14].

Typically the public do not have a direct role in priority setting (i.e. they do not sit on decision-making committees). Their involvement is often limited to consultation via surveys, citizens' juries and advisory committees. However, public participation in priority setting can enhance the legitimacy and fairness of decision making [15], incorporate the views of the community in policy making thus revitalizing the democratic will [16], improve trust and confidence in the health system [17], and improve the quality of decision making [18]. Public participation is "the practice of involving members of the public in the agenda-setting, decision-making, and policy-forming activities of organizations/institutions responsible for policy development [19]." (For the purpose of this paper public participation, public involvement and public engagement will be used interchangeably.)

This study focuses on public engagement in a key priority setting context that plagues every health system around the world: wait list management. Waiting for care in publicly funded systems is recognized by all OECD countries as one of the most important health system problems [20]. However, there is no consensus on how to set wait time targets and prioritize wait lists. Consequently, a legitimate and fair process must be used to address this crucial priority setting problem.

Wait time strategies have been studied at the micro (or bedside) level (e.g. in critical care [21] and cardiac surgery [22]). However, to our knowledge there has not been an empirical study describing and evaluating a formal system-wide wait time strategy using "accountability for reasonableness" with particular attention to public involvement, nor is there guidance for how to involve the public in a wait time management priority setting initiative.

The purpose of this study was to *describe *priority setting in the Ontario Wait Time Strategy (Ontario, Canada) and *evaluate *it with particular attention to public involvement.

## Methods

### Design

To describe the priority setting process of the Ontario Wait Times Strategy (OWTS) we used qualitative case study methods. This is the most appropriate empirical approach because priority setting in healthcare is complex, context dependent and involves social processes [23]. To evaluate the description of the OWTS we used the four conditions of 'accountability for reasonableness' (described below).

### Setting

The OWTS aims to achieve meaningful reductions in wait times in five areas (cancer, cardiac interventions, joint replacement, sight restoration and diagnostic imaging), improve efficiency by which patients needing those services are managed, set appropriate wait time targets, and develop a system to prioritize patients by need.

### Sampling and sample size

We sought to interview all people in leadership positions at the OWTS. All but one agreed to be interviewed. These included OWTS staff, Ontario Ministry of Health and Long Term Care (MoHLTC) staff, expert panel members, and the like. In addition we asked these participants to suggest others who were relevant to the research. As a result we interviewed individuals at the MoHLTC, hospital CEOs, patient advocates, nongovernmental associations and medical associations. Sampling continued until we began to hear the same views repeatedly in consecutive interviews – sometimes called saturation. Sample size was not formally calculated, but sampling decisions were made concurrently with the data analysis and continued until no new concepts arose during the data analysis.

### Data Collection

Data was collected between January 2006 and July 2006. There were two primary sources of data for this case study: (1) documents (e.g. strategic planning reports, public updates), (2) in-depth one-on-one interviews with informants (e.g. OWTS participants, MOHLTC representatives, clinicians, patient advocates). Documents were obtained in electronic form where possible. Documents included: all documents posted on the OWTS website, all pertinent articles published in two major newspapers between and all relevant government press releases. Documents were obtained between January 2006 and July 2007. Documents were selected based on their ability to provide an understanding of the public participation in the OWTS priority setting process and information on how decisions were made, what factors were considered, who was involved in decision-making and how the strategy was disseminated. In total, 25 documents and 28 interviews were collected and analyzed. Those who were interviewed we selected on the basis that they would provide information and insight into the priority setting activities of the OWTS that was not available by public means (i.e. public documents, press releases, etc.). Interviews were audio-taped and transcribed. Initial interview guides were developed based on previous research [9,21,22,24-26] and the conceptual framework, and were revised during data collection in order to pursue emerging findings. The final data set for this study consisted of 25 documents and 28 interviews.

### Data analysis

Data analysis proceeded in three phases: open coding, axial coding, and evaluation. First, in open coding, the data (documents and interview transcripts) were read and then fractured by identifying chunks of data that related to a concept or idea (e.g. equity and media). Second, in axial coding, the concepts were organized into emerging themes that were derived from the data and the four conditions of 'accountability for reasonableness'. Third, evaluation involved comparison between the description of the case study (i.e. what they did) with the conceptual framework (i.e. what they should do). Points of agreement with the framework were considered good practice; points of divergence were marked as areas for improvement [27].

We addressed the validity of our interpretations in four ways. First, two researchers (RAG and DKM) coded the raw data to ensure consistency and accuracy. Second, the emerging findings were presented to an interdisciplinary research team who questioned the analysts. The research team consisted of faculty and graduate students who conduct priority setting research in a variety of contexts. This served to enhance 'reflexivity' and check preconceived assumptions. Third, a rigorous record of the data analysis and methodology was documented to ensure a critical appraisal of the methodology. Fourth, a member check with three leaders at the OWTS verified the verisimilitude of the findings.

### Conceptual Framework

This research was guided by an explicit conceptual framework – 'Accountability for reasonableness' is a conceptual framework for legitimate and fair priority setting[5,6]. It is theoretically grounded in justice theories emphasizing democratic deliberation, thus providing principled guidance for priority setting [28,29]. Also, it has been used to provide practical direction for decision-makers to improve their priority setting processes and operationalize the concept of fairness in their decision processes [8,24].

According to 'accountability for reasonableness' a fair priority setting process meets four conditions: relevance, publicity, revisions/appeals, and enforcement (Figure 2).

### Research Ethics

Research ethics approval was granted from the Sunnybrook Health Sciences Centre Research Ethics Board. Written and informed consent was obtained from all participants. All data and participation were kept strictly confidential and available exclusively to the research team. In dissemination of the research in any form, participants' anonymity has been strictly protected.

## Results

**Figure 1:**
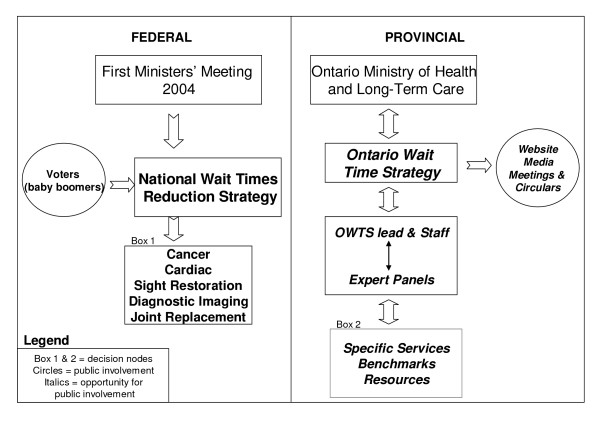
Wait Time Strategy with focus on public engagement.

**Figure 2:**
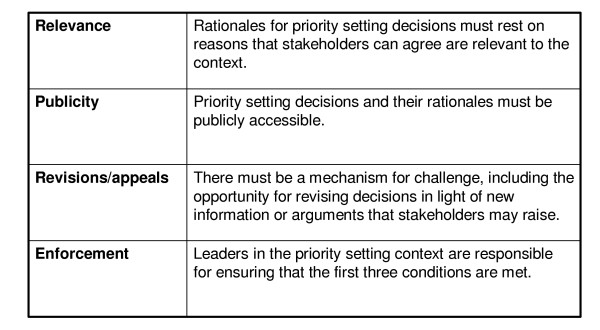
The four conditions of 'accountability for reasonableness'.

In this section we present the findings of our empirical study. This section is organized into two parts: In Part One, we *describe *the Ontario Wait Time Strategy (OWTS) and its broader context (see Figure 1 for a schematic overview of the wait time strategy as it pertains to this study). Specifically we provide a broad overview of the federal wait time initiative and an in-depth description of the OWTS including, the structure of the OWTS, the dissemination efforts of the OWTS, and the priority setting activities of the OWTS; In Part Two, we *evaluate *the wait time strategy, with special attention to public involvement, using the participants’ perspectives and ‘accountability   for reasonableness’ (see Figure 2 for the conceptual frame-work), and describe consequences of the OWTS identified by the study participants.

### Part One – DESCRIPTION

#### The National Wait Times Reduction Strategy

At the 2004 Annual Conference of Federal-Provincial-Territorial Ministers of Health, the Canadian First Ministers agreed to achieve meaningful reductions in wait times in five key areas (cancer, cardiac interventions, joint replacement, sight restoration and diagnostic imaging procedures) by March 31, 2007. The *National Waiting Times Reduction Strategy *was an effort to better manage wait times and achieve a measurable reduction of wait times where they are longer than medically acceptable. Each of Canada's provinces was given the responsibility to markedly decrease wait times in these five areas within their province. To help, the Federal government allocated $5.5 billion over 10 years to the provinces for: hiring more health professionals, clearing backlogs, building capacity for regional centers of excellence, expanding appropriate ambulatory and community programs and developing tools to manage wait times.

#### The Ontario Wait Times Strategy (OWTS)

As a result of the federal wait time initiative, on November 17, 2004 the Ontario Ministry of Health and Long-Term Care (MOHLTC) introduced *The Ontario Wait Times Strategy *(OWTS), to improve access and reduce wait times. Guided by the federal selection of the five wait time areas, the OWTS decided to specifically target: cancer surgery, cardiac revascularization procedures (coronary angiography, percutaneous coronary intervention, and coronary artery bypass graft surgery), cataract surgery, total joint hip and knee replacements, as well as Magnetic Resonance Imaging (MRI) and Computerized Tomography (CT) scans. The MOHLTC allocated $222.5 M (Can) to the strategy.

##### Structure of the OWTS

An experienced clinician-administrator was appointed by the Ontario Minister of Health to lead the OWTS, and he assembled and supervised the work of 'Expert Panels' that addressed each of the OWTS focus areas. The panels were created to consult on access to care and wait times in the five areas, and consisted of clinicians, administrators, researchers, informatics personnel and relevant health system leaders – some panels included hospital CEOs and one panel included a NGO Director. There were no public (or lay) representatives on the panels. As shown in the expert panel reports, the panels identified strategies to increase system capacity, reduce wait times, and prioritize patients, and provided advice on best practice targets, human resources, current and emerging technologies, funding, information management, quality standards, and future needs. All panel reports were reviewed by OWTS leaders and ultimately delivered to the Minister of Health.

##### Dissemination to the Public

The OWTS made an effort to disseminate their actions to the public, as evidenced by their construction of a wait times website and associated advertisement campaign, as well as their use of media briefings. The OWTS launched a public website in December 2004 that reported on the actions and plans of the strategy and allowed the public to search wait times according to procedures in varying geographic locations across the province. All panel reports have been published on the website within 10 working days of their receipt. To promote awareness of the website the government launched an advertisement campaign: "*It's Worth Knowing*". From the analysis of the interviews it was evident that the media has been instrumental in informing the public about the OWTS – the strategy's leader held media briefings in various cities across Ontario, multiple press releases about the OWTS have been issued by the MOHLTC, the OWTS has published five issues of its newsletter ("wait time updates" – providing information on the actions and progress of the strategy), and OWTS representatives have met with hospital boards and regional health system leaders. Although the OWTS made substantial efforts to disseminate the activities of the strategy the actual decision making ('how' and 'why') were not made accessible to the public.

##### Priority Setting Activities of the OWTS

As the OWTS is part of a federal-provincial-territorial initiative, certain priority setting decisions were made at a national level including the decision to target wait times and the selection of the five focus areas. In addition, the federal government decided on the amount of money allocated to the provinces for wait times. The OWTS did not formally evaluate the legitimacy and fairness of its priority setting activities. There was no formal appeals mechanism for the OWTS priority setting. All feedback from advisory panel members was managed informally by the panel chairs. Any public feedback received from either the MoHLTC email address or media briefings was dealt with informally.

##### The OWTS was responsible for six specific priority setting decisions (see Figure 3)

**Figure 3:**
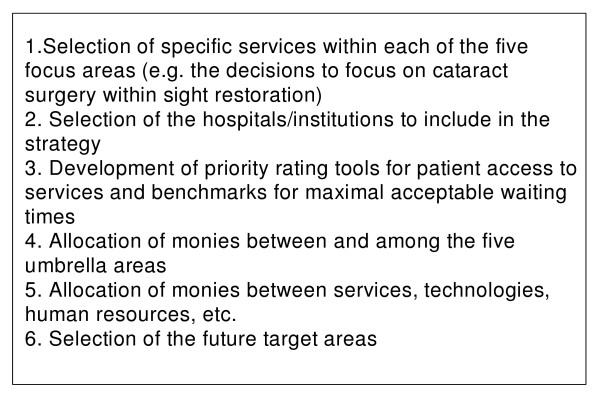
OWTS Priority Setting Decision Nodes.

The OWTS did not set stringent binding criteria to guide its priority setting. The participants of the OWTS that we interviewed described a cluster of eight context-specific factors that they considered in making their recommendations, including: 1) *Capacity *to increase services was a strong determinant in procedure/surgery volume allocations. 2) Because of *time frame *restrictions in which results were expected from the strategy, resources were initially allocated based on a short term, quick fix solution. 3) There were strong efforts to be *consistent *in the planning and implementation of the strategy across the five areas. For example, the OWTS employed one individual to bring a commonality and rigor to the panels deliberations and written recommendations and to avoid conflicting advice. 4) A main goal was to create *equity *between geographic regions and provide equal access to care across the province. 5) Centers of excellence who provided *quality *care were prioritized for funding. 6) The ministry used *efficiency *ratings as part of their criteria for allocating funds – centres of care which provide services efficiently were rewarded with funding for additional cases. 7) When possible, recommendations and benchmarks were based on *scientific evidence*. 8) Expert panel recommendations were partly based on perceptions of *practicality/probability of success*.

These eight criteria were not set by the OWTS – there were no formal guidelines to direct the allocation of funds required by the OWTS. These criteria were loosely used to guide the recommendations of the expert panels and the final priority setting decisions. However, as the criteria were not binding, nor were they set by the OWTS, it is difficult to determine how these factors were weighed in setting priorities. In interviews, some expert panel members emphasized certain criteria more than others. These criteria were not disseminated to the public and the public were not involved in their development.

### Part Two – EVALUATION

In this section we present an evaluation of the wait time strategy, with special attention to public engagement, in two ways: 1) using the participants' perspectives, and 2) using the four conditions of 'accountability for reasonableness.' Also, we describe three consequences of the OWTS as identified by the study participants. Verbatim quotes are included to illustrate key ideas.

#### 1) Public Involvement Evaluated Using the Participants' Perspectives

This section provides an evaluation of the public involvement, according to participants' perspectives. First is a brief analysis of the public involvement in the National Wait Times Reduction Strategy. Second is an in-depth evaluation of public involvement in the OWTS, which includes: a) were the public involved? b) should the public be involved?, and c) how should the public be involved?

##### Public Involvement in the National Wait Times Reduction Strategy

The National Wait Time Strategy did not formally involve the public in their priority setting activities. Some of those interviewed indicated that public input provided the impetus for the wait time strategy itself and the selection of the five focus areas – i.e. the five areas were chosen based on what was perceived to be areas of concern for Canadians, particularly voters between age 45 and 65.

However, there is no concrete documentary evidence to support the notion that the public selected the five areas. Some participants recounted anecdotes about the public's views. The notion seems to have stemmed from informal communications between various politicians and a few constituents, and attention given by the media.

"In terms of hard data there wasn't because there is hard data on none of it. In an ideal world we would have had some sort of data on all surgeries or all medical procedures and been able to say these are the one's in which the wait times are the worst. I think it was principally anecdotal. Certainly in terms of responding to correspondence it is evident to me still that the number one thing people write in about is – I've been waiting for a hip or knee surgery. So there was a rough feeling about gauging the public upset was around these areas."

Public opinion surveys of that time period support wait times as an issue in general [30], but did not identify the five specific areas.

"I think you've probably figured out the 5 came off the top of somebody's head somewhere and they were fundamentally viewed by politicians as public issues and that was the extent of the thinking."

Some of the interviewees said that the choice of the five areas was exclusively politically driven.

"I think most people who are involved in this identified a basket of reasons why these 5 things were picked. They're issues that are relevant to the current politically-powerful demographic, which is the ageing baby-boomers. So, it's cardiac, hips, eyes, it's stuff like that. There's a huge big powerful political group there, who are all in their late 50s, early 60s, and they all need hips and eyes and heart surgery and stuff like that. That's why that stuff got picked."

##### Public Involvement in the Ontario Wait Time Strategy

###### a) Were the public involved?

The public has not been involved in any decisions of the OWTS. Public involvement has been limited to the dissemination efforts of the strategy (i.e. the OWTS website and media briefings). OWTS leaders regarded the website as a pivotal piece in their communication strategy and as the strategy's primary vehicle of public involvement. The website served as a one-way information provider – it did not solicit public feedback.

"I think it's an essential tool. I don't think we can not report the information publicly on the web. I think it's just a basic fundamental thing. I don't think it necessarily means that we can wash our hands of it and say we've told the public everything they need to know....If you look at public involvement as a continuum, that's at the very low end of the continuum. It's simply to inform, so that you have a point for access to information. It isn't really a website that encourages consultation and feedback."

The OWTS has not solicited feedback from the public, nor engaged in dialogue with the public. Ad hoc comments from the general public pertaining to the layout and features of the website have informally influenced the website's design. At the time of this writing, the OWTS planned to conduct focus groups with the public to explore the usability of the website.

"I don't think there has been a strong sense that, for example, expert panels need to add a member of the public or should we put together a panel of individuals from the public and begin to reflect your views of how we should be prioritizing. I haven't heard that a lot. I've heard more of: Are we getting at what we think the public wants?"

###### b) Should the public be involved?

The vast majority of the interviewees did not identify lack of public participation as a short-coming of the OWTS decision making process.

"So public input into it now or in the future is totally acceptable and I think its also based on consensus development with other provinces and so its bound to happen and its totally acceptable... [But,] I am not sure if it is a huge deficiency."

Some participants stated that public involvement would not have influenced the decision-making.

"I'm not sure quite frankly our recommendations would have been changed that much because there was a lot of discussion about the scientific support about wait times and then the psychological impact of wait times."

However, a few participants expressed the view that public involvement would enhance the legitimacy and fairness of the decision making

"On the other hand from a process point of view, from a transparency point of view, it is probably the deficiency in the process."

###### c) How should the public be involved?

The majority of study participants were open to some form of public involvement in the OWTS, however no one methodology was preferred by the majority. Many options were suggested, including: focus groups, surveys, phone interviews, deliberative dialogue, elections/voting, stakeholder roundtable, and development of an arms length public commission on health. Also, they identified a number of opportunities for public involvement in the OWTS, including: identifying priorities, setting benchmarks, decision making within panels, and the selection of targeted services areas.

"By engaging the public. [That is] how it can be improved. By being open to know what's driving all of your decisions to prioritize five areas of the health system. In a hydraulic system you are doing so at the expenses of something else. You have to convince the public that this is really worth doing – That a new hip is worth much more than preventing a heart attack, or preventing amputation. That a new cataract is worth much more that overcoming a crippling or severe debilitating depression. That you have the moral high ground to make those decisions. That depression is less important than a cataract. If you can do that then you have risen to the moral high ground. If you have that ability to do that, then you've done a great job. But if you can't convince the public that depression isn't worth less than a cataract, then you have to talk with them."

Moreover, participants suggested that there should be public involvement regarding more general priority setting issues and the sustainability of the healthcare system, and where and why recourses should be allocated within healthcare. As a result of the finite resources available for healthcare many participants expressed a need for public input in setting priorities in health.

"All of this is political isn't it. We really need a good dialogue by Canadians about what they want."

"Once you actually have good info then the public can be more involved in allocation priorities, because when you have a scarce resource and there is choices being made and it is a public system the public needs to be involved in how those choices are being made."

##### Benefits and Concerns with Public Involvement

Participants described their thoughts on the benefits and concerns of involving the public in the wait time strategy. Not all participants shared the view that public involvement would benefit the strategy nor were all participants concerned with the idea of public involvement. However, the benefits and concerns that were identified fell into four categories of benefits and four categories of concerns. The participants' views about public involvement in the OWTS included four benefits and four concerns. (These are summarized in Table 1.)

**Table 1:** Benefits and Concerns Regarding Public Involvement in OWTS

**Benefits**	**Concerns**
1. Supporting the initiative	1. The public are biased
2. Experts of the lived experience	2. The public are not being involved
3. A publicly funded system	3. Time constraints do not permit public involvement
4. Sustainability of the healthcare system	4. The public are not informed sufficiently for the level of technicality.

###### Benefits

####### a. Supporting the initiative

Without public input, priority setting decisions lack buy-in and support from the largest and most important stakeholder group. The OWTS will likely be more successful and meet less resistance by involving the public.

"Well, attempting to set priorities that doesn't involve public input is almost certainly going to fail, because again, you have a finite resource and infinite demand. So without very secure support from a large enough segment of the population for a specific initiative, it will fail."

" [By not involving the public] I think it is a very poor approach to achieve system change because it doesn't engage the people to develop buy-in."

####### b. Experts of the lived experience

Although the majority of participants viewed the priority setting as a technical exercise, a few identified the need to consider the public's lived experience with illness and the health system.

"Patients are the ones that tell you what the real impact of waiting is.... Even if a patient is relatively low risk on one of those events, there is still a downside of waiting too long: social impact, personal/psychological impact, financial impact, quality-of-life impact in general. You know, they're hard to measure and they don't show up through medical exams. And it's only the public that tells you about that.... So I think the public has a lot to contribute about the general impact of wait time, but also just in forcing ourselves to explain it to patients better, it forces us to look at the process more. So that's sort of the dual role that the public has to play here."

####### c. A publicly funded system

Some participants argued that because the health system is publicly funded, the public should be involved in the OWTS.

"This is a publicly funded health system. The public have a right to know; the public have a right to get back to us; the public have a right to assert themselves into the process; and the more they are involved, the more they are ingrained, the stronger the system we have."

"See, this is the interesting piece, right, you own our system, because you pay for it out of your tax dollars and so, you should at least have a say at some point in this journey...."

####### d. Sustainability of the healthcare system

Participants recognized that, because there are increasing demands on the health system and finite resources, in order to sustain a publicly funded system public consultation must occur about which services should be covered and which initiatives (i.e. the OWTS) should be funded.

"I think that the public does have a role very much in sustaining the strategy.... I think that the public, when it comes to sustainability and the whole wait time strategy, needs to get more involved and I don't know how this would happen, needs to get more involved in those discussions about limits to what medicine can do."

###### Concerns

####### a. The public are biased

Amongst the interviewees there was a strong belief, as evidenced by their emphatic statements, that most public participants would be strongly influenced by their personal values and beliefs and would not be able to contribute objectively to the OWTS priority setting activities.

"I don't disagree that the public should be engaged....However, the public makes decisions based on multiples of 1, so I make decisions based on my experience and I tell you what I need based on my personal experience. That can be somewhat dangerous, I think."

"One of the issues ....was that you know its really really hard to have a patient, whatever you want to call it, which isn't' bound by an organization, or ethical policies, or all of those things, to have them at the table and engaging at a level beyond "this impacts on me" and its me we're really here to talk about. And I fully understand that. Having said that, I would suggest that there are groups of consumer experts out there who are not talking that role."

####### b. The public are not interested in being involved

A great number of those interviewed stated that the public do not want to be involved in the priority setting activities of the OWTS.

"I guess in my own view the public wants to be a consumer they don't want to direct the system....so my own view is as a consumer I don't want to be involved in telling you how to fix the system, just fix it....My own view is just very much towards, in this day and age people are consumers. You know when you go to McDonalds you don't really give a damn how they get your hamburger there in three minutes you know."

"I seem to recall reading ....about the willingness of the public to participate in these kinds of things. And my understanding is it's relatively limited. People want, at the end of the day, my neighbour, my mother, whatever, I want to know that the service is there when I will need it. I don't want to get involved in the debates or the decisions about how that happens."

####### c. Time constraints do not permit public involvement

Participants explained that a major reason for not involving the public is lack of time because the OWTS was heavily driven by deadlines. Consequently, many said that there was no opportunity for public involvement.

"We did talk about it but the reason we didn't [involve the public] is because of the speed in which we had to complete this. We had basically five weeks to complete the process so we didn't actually have a lot of time for public consultation."

"We are doing this whole thing [on] a terrifically tight program here which has advantages and disadvantages. We hit every mark on time on budget, which is very very tight. So things where you say where is the discussion, there isn't time for it."

####### d. The public are not informed sufficiently for the level of technicality

Many of the participants expressed the belief that the public should not be involved in the OWTS priority setting because decisions were very technical and public representatives would not be able to contribute.

"I really don't [see a role for the public] on my panel, because its so sort of, its technical."

"All those kinds of things that were in the expert panels' reports, I'm not sure that taking a member of the public that isn't very familiar with health care, I don't think he or she would've contributed much to those kinds of discussions and decisions. And they probably would not have been very comfortable being in that kind of forum, ...I don't think that that would've been a good use of their time. "

#### 2) Evaluation of the OWTS Priority Setting Process Using 'Accountability for Reasonableness'

The four conditions of 'accountability for reasonableness' (Figure 2) can be used to evaluate the legitimacy and fairness of the OWTS. Elements that comply with the framework can be considered examples of 'good' practice, while divergence with the framework can help identify opportunities for improvement.

##### Relevance

Efforts were made to base OWTS decisions on justifiable factors, including scientific evidence, and most panels used similar rationales. However, there was no attempt to explicitly articulate or consistently apply these rationales, either between different decisions or decision makers. The public were not involved in any of the strategy's decision making, though many other stakeholders were. Consequently, there was no way of knowing whether the 'experts' identified relevant reasons, or applied them, in the same way as the public would. The Relevance condition was only partially satisfied.

##### Publicity Condition

There was a deliberative effort by leaders in both the MOHLTC and OWTS to be transparent about the strategy. All expert panel reports were posted on the OWTS website within 10 working days of their receipt. The OWTS also disseminated the activities of the strategy to the public in a number of ways, including "wait time updates", press releases, scholarly papers, meetings with hospital and regional leaders, and media briefings. However, the actual decision making ('how' and 'why'), was not made accessible – the cluster of considerations were only identified through this study. The publicity condition was only partially met.

##### Revision/Appeals Condition

There was no formal appeal mechanism for the OWTS. The public were not provided an opportunity to challenge the decision making criteria or rationales. Feedback within each advisory panel was managed informally by the panel chair. When the OWTS team received feedback at meetings and media briefings, it was dealt with informally. The OWTS has planned to conduct focus groups with members of the public to obtain feedback on the usefulness of the website. The Revision/Appeals condition was not met.

##### Enforcement Condition

Leaders in the OWTS have attempted to ensure transparency, stakeholder involvement, and that decisions were based on justifiable rationales. However, the rationales for specific decisions were not disseminated and the public were excluded from the OWTS. Moreover, the OWTS has not formally evaluated the legitimacy and fairness of its activities. The Enforcement condition was only partially met.

#### Consequences of the OWTS

Although our study did not seek to explore the consequences of the OWTS, many participants voluntarily expressed concern for the consequence of the strategy, and suggested that more time and effort should be spent monitoring the consequences.

"I don't think that we ever really sat down, myself included, and looked at the unintended consequences or collateral damage that might occur with the Wait List strategy."

Three main consequences were identified by interview participants, including:

1) Cannibalism – some participants expressed concern that operating theatre time and clinicians' time was being dominated by the wait time strategy, and attention to other service areas (e.g. paediatrics, mental health) was suffering.

"We are having 17–20 ORs cancelled a month because we can't compete with access to resources that are required to meet targets. And so, these are given priority so that means that both streams, if you will, or for that matter, things coming through the emerg., anybody else who needs the OR, who needs the anesthetists, the nurses and other staff to make surgeries happen is disadvantaged."

"The health system is a hydraulic system, it is closed hydraulic system, if you push down on one point it comes up at a different point. And once you prioritize one part of the system, I don't have a very good word for it, you cannibalize some other part of the system."

While many said they believed that cannibalism was happening, others were adamant that the OWTS has taken measures to prevent cannibalism. They believe that since there is dedicated funding for the OWTS services, funding to other areas will not be compromised. One participant stated that a data set of Ontario Health Insurance Plan billing did not indicate that other services had been affected by the OWTS. Additionally, the MOHLTC stipulated in hospital accountability agreements that other service areas must not be jeopardized.

2) Sustainability – participants expressed concern for the strategy's sustainability because of limits to resources (financial and human) and concern that decision-making was driven by short-term solutions. Many verbalized the opinion that the current government was overly focused on re-election and using reduced wait-times in the five targeted areas as a key plank in their political platform, at the expense of longer-term strategic improvements to the health system. Some participants articulate concern for the sustainability of the OWTS.

"The go forward directions need to be thought about in a more system way than this targeted approach which I think was really designed as a boutiques way of showing that if you target an effort you achieve the result that you want to achieve. And for a short time I think you can. I think its achieving sustainability that is going to be more difficult."

3) Two Class System – as a result of the increased funding directed at five service areas, participants expressed concerned that two classes of patients were being created – that is, those who are part of the wait time strategy were receiving better care and better access to care.

"The other thing is, we've created 2 tiers of patients, whether we intended to or not, Ok. And not only have we put divisions against each other, we put surgeons within divisions against each other i.e., if you're an orthopedic surgeon who doesn't do joints, then your income, your livelihood and your patients are now less."

Our primary reason for including participants' views about these three consequences was that the participants specifically identified these as important reasons why the public should be involved in the priority setting of the wait time strategy.

## Discussion

To our knowledge, this is the first time a formal system-wide wait list initiative has been described and evaluated with a specific focus on public involvement. Therefore, new lessons can be gleaned from this study, such as to how to enhance the legitimacy and fairness of priority setting and where and how the public can be engaged in the decision-making (please see Results: Part Two – evaluation). These findings will almost surely be helpful to similar wait time initiatives elsewhere. Leaders in wait time initiatives can gain insight into the how, why and where to involve the public in priority setting. The concrete steps for involving the public can be applied to most wait time initiatives. Although it has been established in the literature that public engagement is laudable, there is a gap as to how to practically involve the public in a wait time management initiative. This study fills such gap. A key strength of this study is its grounding in an explicit conceptual framework – 'Accountability for reasonableness' not only provides a useful framework for evaluating the legitimacy and fairness of any priority setting initiative, it also provides the theoretical and practical platform upon which to ground an evaluation of public involvement.

Figure 1 provides an overall schematic of the wait time effort in Canada as it pertains to this study, indicating public involvement efforts to date and suggestions for where to enhance public involvement. As discussed in the introduction public engagement will bolster the priority setting of the OWTS – it will enhance the legitimacy and fairness of the priority setting[15]; enhance trust and confidence in the health system overall[17]; improve the quality of decision-making[18]; and strengthen the sustainability of the OWTS[31].

There is ample opportunity to engage the public in the priority setting activities of the OWTS. Although many priority setting decisions have been made in regards to the OWTS, it is an ongoing initiative which continues set priorities. Table 2 provides a brief overview for how to operationalize a public involvement strategy. There is much to be learned from other examples of priority setting initiatives which have successfully implemented similar strategies (i.e. National Institute for Health and Clinical Excellence Citizens Council, Canadian Expert Drug Advisory Committee, and the Citizens' Council for Drug Policy in Ontario). The proposed plan is based on this data analysis, current public involvement literature, and guided by the 'accountability for reasonableness' framework, it includes: 1) *Shared Decision-Making *– collaboration between the public and 'experts' will enhance legitimacy and fairness at all stages of OWTS decision making; 2) *Focused Outreach *– use public consultation techniques to help determine which health services should be part of the OWTS, and consult the public on the lived experience of using the health system to aid in the development and refinement of priority rating tools and benchmarks; and 3) *Feedback and Appeals Mechanism *– a formal mechanism, with channels to decision makers, to permit public feedback on priority setting activities will enhance the responsiveness in the strategy. The aforementioned plan was presented to the OWTS and Ontario Ministry of Health and Long-Term Care (MoHLTC). It received much accolade. At the time of this writing the research team was in the process of working along with the OWTS to implement the three components of the plan. Although not all participants endorsed public engagement, as evidenced in the results section, it is a necessary component of fair and legitimate priority setting. At the time of this writing the MoHLTC were receptive to implementing the proposed public engagement plan. Accordingly it is imperative for the MoHLTC to strive to mitigate these dissenting attitudes and take the necessary steps to help integrate public involvement in decision making and encourage receptiveness of public involvement in priority setting.

**Table 2:** A Public Involvement Strategy for the Ontario Wait Time Strategy

Public Involvement Foci	Operational Plan
Shared Decision-making	1) Create positions for public members on the expert panels → assign public positions on each panel → provide a workshop to educate citizens and experts of their roles and responsibilities
	2) Construct a Citizen's Panel, consisting of the assembled public members from the expert panels, to collaborate with the OWTS and provide ongoing advice on priority setting (The Council can also act as an advisory body for other areas of health care priority setting, i.e. wait times, drugs, mental health, etc.) → provide education for citizens and experts pertaining to their responsibilities and mandate → present council with information on priority options and their consequences – have them consider how to prioritize options → consider broad health system priorities – i.e. should better chronic disease management be a priority over waiting lists

Focused Outreach	1) Conduct deliberate polling exercises with the purpose of determining which health service areas should be included in the OWTS → this information will be used to help the OWTS select future target services and areas of focus
	2) Conduct a series of focus groups with the purpose of understanding the public's views on the priority rating tools and benchmarks → this information will be used to help refine the current and develop new priority rating tools and benchmarks → some existing benchmarks have accounted for the presumed psychological impact of waiting – concrete public consultation regarding the lived experience will allow the psychological burden of waiting to be properly accounted for in the benchmarks

Feedback and Appeals Mechanism	1) Create a feedback section on the OWTS website to solicit general feedback on the priority setting activities

Some participants in this study cited four concerns about public involvement (see Table 1). These concerns are all too familiar disputes of public involvement in policy making, and are easily countered:

*Concern #1: *The public are biased. *Response: *Members of the public are no more biased than others traditionally involved in priority setting decisions, including administrators, scientists and clinicians. Moreover, priority setting decisions are not technical and objective, but value-based, subjective, and they require deliberation.

*Concern #2: *The public are not interested in being involved. *Response*: Traditionally, priority setting has been framed as a technical exercise, involving the evaluation of scientific evidence and economic analyses. It is self-evident that members of the public would be reluctant to participate in technical discussion for which they are not equipped. However, at its core, priority setting involves the adjudication of values – e.g. When should we preferentially fund geriatric services over paediatrics? – for which the public is prepared. Moreover, multiple studies have shown the public's willingness to participate in these complex and difficult policy decisions[25,26,32].

*Concern #3: *Time constraints do not permit public involvement. *Response: *There are three benefits in taking time to engage the public. First, a speedy process does not always result in quality outcomes – engaging the public will enhance the quality of priority setting decisions. Second, if the necessary time is not taken to ensure the legitimacy of decisions and to obtain stakeholder buy-in, a huge amount of time is spent afterwards addressing the objections and conflicts[33]. Third, establishing an ongoing culture of public engagement, with embedded mechanisms, would alleviate the concern over time.

*Concern#4: *The public are not informed sufficiently for the level of technicality. *Response: *Priority setting involves value-based decisions, and members of the public offer insight into the values and beliefs of the public at large. Moreover, members of the public are experts regarding the lived experience of using the health system.

Through this evaluation we have identified good practices and opportunities for improving the legitimacy and fairness of the OWTS. A significant improvement would come through involving the public, the largest and most important stakeholder group in any health system. Involving the public will enhance the legitimacy and fairness of the OWTS, which are the key goals in priority setting. Public engagement leads to a better understanding of policy decision making and thus increased trust and confidence in the health system [6,16,34-36]. As well public engagement will improve the quality of decision making. Priority decisions are value laden and affect the fundamental wellbeing of society, and members of the public are the 'experts' in identifying and weighing the values of their society[37,38]. Clinicians' perspectives and medical expertise are imperative for priority setting, but they do not and can not replace the views of the public – involving the public directly in priority setting decision making will bridge the gap between misconceptions and authentic values[31,39]. Moreover, public involvement provides another layer of scrutiny to the process, which will help ensure a higher quality of decision.

### Limitations

The primary limitation of this study is that the findings may not be entirely generalizable to other contexts. However, generalizability was not a goal of this study. Nonetheless, it is highly likely that lessons from our study will be helpful to others. Moreover, the approach we have used here (describing a case and evaluating it against 'accountability for reasonableness'), can be considered a key part of meeting the leadership requirements for legitimate and fair priority setting. These methods can be duplicated with great benefits in any other priority setting context [27]. Second, this study is time limited – initiated in January and concluded in July 2006. The OWTS is an ongoing and dynamic initiative, which is continuing to learn and revise their strategy. However, the majority of key priority setting decisions pertaining to the OWTS were made during the period of this study. The third limitation is social desirability bias – interviewees' views may reflect what they thought the researchers wanted to hear. However, the parallel analysis of documents provides verification for the interview data.

## Conclusion

This study helps to address three gaps in the scientific literature. Firsts, we have provided an in-depth description of real world priority setting in wait time management with a focus on public engagement. Second, we have evaluated the description of priority setting in a wait time management initiative using 'accountability for reasonableness', and have identified both areas of good practice and opportunities for improvement which will be helpful to other decision-makers in comparable priority setting endeavors. Third, from this study we have developed practical guidance for when and how to engage the public in wait time management priority setting. Our approach – describe, evaluate, improve – can be used as a helpful learning platform for others engaged in priority setting.

## Competing interests

The author(s) declare that they have no competing interests.

## Authors' contributions

RAB conducted the data collection (interviews and documents) on which this paper is based, collated and analyzed the data, and drafted the manuscript.

DKM participated in analyzing the data and commented on earlier drafts of the manuscript and was involved in revising it critically for important intellectual content.

WL commented on earlier drafts of the paper.

AL commented on earlier drafts of the paper.

All authors made substantial contributions to the conception and design of the study and read and approved the final manuscript.

## Pre-publication history

The pre-publication history for this paper can be accessed here:



## References

[B1] OECD (2006). Rising health costs put pressure on public finances.

[B2] Ham C (1993). Rationing in Action: Priority Setting in the NHS: Reports from Six Districts. British Medical Journal.

[B3] Klein R (1993). Dimensions of Rationing: who should do what?. British Medical Journal.

[B4] Holm S (1998). The second phase of priority setting. Goodbye to the simple solutions: the second phase of priority setting in health care. British Medical Journal.

[B5] Daniels N, Sabin J (2002). Setting Limits Fairly: Can we learn to share medical resources?.

[B6] Ham C, Robert G, eds (2003). Reasonable Rationing: international experience of priority setting in health care.

[B7] Daniels N (2000). Accountability for reasonableness. British Medical Journal.

[B8] Ham C, Coulter A, eds (2000). Global Challenges of Health Care Rationing.

[B9] Martin DK, Shulman K, Santiago-Sorrell P, Singer PA (2003). Priority Setting and Hospital Strategic Planning: A Qualitative Case Study. Journal of Health Services Research & Policy.

[B10] Jordan J, Downswell T, Harrison S, Lilford RJ, Mort M (1998). Health needs assessment: whose priorities? Listening to users and the public. British Medical Journal.

[B11] Charles C, DeMaio S (1993). Lay participation in health care decision making: a conceptual framework. Journal of Health Politics, Policy and Law.

[B12] Daniels N, Sabin JE (1997). Limits to health care: fair procedures, democratic deliberation and the legitimacy problem for insurers. Philosophy and Public Affairs.

[B13] Department of Health (1992). The patient's charter.

[B14] Ontario Ministry of Health (1989). Deciding the future of our health care: an overview of areas for public discussion.

[B15] Fleck LM (1994). Just Caring: Oregon, healthcare rationing and informed democratic deliberation. Journal of Medicine and Philosophy.

[B16] Coote A, New B (1997). Direct public and patient involvement in rationing. Rationing Talk and Action in Health Care.

[B17] Lengahan J, Hunter D (1997). Rationing Health care: the political perspective. British Medical Bulletin.

[B18] Ham C (1996). Rationing in Action: Priority Setting in the NHS: Reports from Six Districts. British Medical Journal.

[B19] Rowe G, Frewer L (2005). A Typology of Public Engagement Mechanisms. Science.

[B20] Hurst J, Siciliani L (2003). OECD Health Working Papers: Tackling Excessive Waiting Times for Elective Surgery: A Comparison of Policies in Twelve OECD Countries.

[B21] Mielke J, Martin DK, Singer PA (2003). Priority Setting in Critical Care: a Qualitative Case Study. Critical Care Medicine.

[B22] Walton N, Martin DK, Peter E, Pringle D, Singer PA (2006). Priority setting in cardiac surgery: A qualitative study. Health Policy.

[B23] Strauss A, Corbin J (1998). Basics of Qualitative Research: Techniques and Procedures of Developing Grounded Theory.

[B24] Gibson JL, Martin DK, Singer PA (2001). Priority setting for new technologies in medicine: a transdisciplinary study. BioMed Central Health Services Research.

[B25] Kapiriri L, Norheim OF, Heggenhougen K (2003). Public participation in health planning and priority setting at the district level in Uganda. Health Policy and Planning.

[B26] Singer PA, Martin DK, Giacomini M, Purdy L (2000). "Priority Setting for New Technologies in Medicine: A Qualitative Case Study". British Medical Journal.

[B27] Martin DK, Singer PA (2003). Strategy to Improve Priority Setting in Health Care Institutions. Health Care Analysis.

[B28] Cohen J (1994). Pluralism and Proceduralism. Chicago-Kent Law Review.

[B29] Rawls J (1993). Political Liberalism.

[B30] Update #1 The Wait Time Strategy.

[B31] Rifkin SB (1996). Paradigms lost: toward a new understanding of community participation in health programmes. Acta Tropica.

[B32] Mossialos E, King D (1999). Citizens and Rationing: analysis of a European survey. Health Policy.

[B33] O'Hara K (1998). Citizen Engagement in the Social Union. Securing the Social Union.

[B34] Traulsen JM, Almarsdottir B (2005). Pharmaceutical policy and the lay public. Pharmacy World & Science.

[B35] Ham C, McIvers S (2000). Contested decisions: priority setting in the NHS.

[B36] Ham C, Pickard S (1998). Tragic choices in healthcare: the case of child B.

[B37] Beierle TC (1999). Using social goals to evaluate public participation in environmental decisions. Policy Studies Review.

[B38] Tenbensel T (2002). Interpreting public input into priority-setting: the role of mediating institutions. Health Policy.

[B39] Heritage S (1994). Community participation in primary care.

